# Supplementary material for: Retinal Toxicity Induced by Chemical Agents
Source: Int J Mol Sci. 2022 Jul 25;23(15):8182. doi: 10.3390/ijms23158182 (PMC9331776; doi:10.3390/ijms23158182)
Supplement: Supplementary file 1 [file ijms-23-08182-s001.zip › ijms-1807709-supplementary.pdf]

Supplementary material:

To prepare this review we mainly use the Pubmed database as a tool to search all the studies. For natural products, the search began with the studies retrieved by the following keywords and combinations: *medicinal herbs, retina; medicinal plant, retina*.

Then we refine our research by using the combination of *retina* and *the specific name of the plant/herb/natural compound/isolated molecule* (more than 100 compounds). The research with the following words gave zero results, indicating no published study in the retina: *Acacia nilotica*; *Actaea racemosa*; *Amophorphallus konjac*; *Anciodus tanguticus*; *Andrographis paniculata*; Caralluma; Capsicum annuum L; *Cynara cardunculus*; *Cynara scolymus*; Diallyl monosulfide; Diallyl trisulfide; *Echinacea purpurea*; *Equisetum arvense*; *Foeniculum vulgare*; Glucomannan; *Harpagophytum procumbens*; Ligusticum chuanxiong Hort; *Lippia sidoides*; *Matricaria chamomilla*; *Maytenus ilicifolia*; *Mentha piperita*; *Mikania glomerata*; mint; *Paullinia cupana*; *Peumus boldus*; phaseolamine; *Phaseolus vulgaris*; *Pholia magra*; piperita; Porangaba; *Psidium guajava*; *Rhamnus purshiana*; *Salix alba*; *Schinus terebinthifolia*; *Serenoa repens*; Sipjeondaebo-Tang; Slendeta; *Stryphnodendron adstringens*; *Trifolium pratense*; *Uncaria tomentosa*; *Valeriana officinalis*.

Moreover, the research with *retina* and several *other molecules/plants/herbs/natural compounds*', listed ahead, results in one or more papers showing a protective role: *Acer palmatum thumb; Aesculus hippocastanum*; *Acokanthera oblongifolia*; aloe-emodin; *Apigenin-7-diglucuronide; Arctium Lappa (arctiin);* Astaxanthin; *Astragalis radix; Astragalus membranaceus*; berberine; bilberry; blueberry; Brazilian Green Propolis; caffeic acid; *Calendula officinalis*; *Camellia sinensis*; *Centella asiática*; *Cinnamomun zeylanicum; Cladophora glomerate*; *Coreopsis tinctoria;* Crocetin; curviflorus; *Dendrobium chrysotoxum*; epicatechin; *Epigallocatechin Gallate*; *Fructus lycii;* Fufang xueshuantong; *Garcinia cambogia*; garlic; *Ginkgo biloba*; Ginseng; Green tea; *Gymnema montanum*; hesperetin; *Hypericum perforatum*; Litsea japonica; Lupeol; *Lycium barbarum*; Menta-FX (a mixture of Panax quinquefolius L.); methyl tyramine; *Myrtus communis;* Notoginsenoside R1 (NGR1 - *Panax notoginseng*); *Passiflora incarnata*; *Plantago asiatica*; *Plicosepalus curviflorus; Polygonum cuspidatum*; *Puerariae radix;* rescinnamine; S-allyl L-cysteine (an active component of garlic); saffron; *Salvia miltiorrhiza*; *Saussurea lappa* (lappa)*; Scolymus hispanicus;* scutellarin; soybean; soy isoflavone; Spirulina; Tangningtongluo*;* Tetramethylpyrazine; *Thymus vulgaris;* Trigonella foenum-graecum (fenugreek); ursolic acid; *Urtica dioica*; vitamin C and E; *Zingiber officinale*; *Zingiber zerumbet*.

Finally, only a few studies showing damage of the neural retina or visual function by natural products were found. The toxic agents were: Diallyl disulfide (DADS); *Embelia ribes* and *Hagenia abyssinica*, two naturally occurring anthelmintics; Hypericin and Kava kava extract (KKE).

The articles selected for the pesticides section were searched in the Pubmed database using the following combination of words: "pesticides and retina". The search was refined using the combination of *retina* and the *name of the main pesticides used in Brazil, the United States and the European Union*. Searching for the following words yielded zero results, indicating no published studies evaluated the retina: *Atrazine*, *Metolachlor-S*, *Dichloropropene*, *2,4-D*, *Metam*, *Acetochlor*, *Metam Potassium*, *Chloropicrin*, *Chlorothalonil*, *Pendimethalin*, *Ethephon*, *Mancozeb*, *Metolachlor*, *Hydrated Lime*, *Propanil*, *Dicamba, Trifluralin*, *Decan-1-ol*, *Copper Hydroxide*, *Acephate*, *Methyl Bromide*, *Glufosinate*. On the other hand, the search combining retina and the following terms yielded results: *Chlorpyrifos*, *Cypermethrin*, *lefenuron*, *thiamethoxam*, *Glyphosate*, *Triphenyltin*, *Thiram*, *Benzimidazole*, *Hypericin*. Although they are pesticides, *6-hydroxydopamine* and *Paraquat* were disregarded as they are a well-established experimental model for neurodegeneration. For the text on pesticides, only scientific articles published between 2000 and 2022 that related the different

pesticides with retinal damage were considered. Pesticides were subdivided into insecticides, herbicides and fungicides.

The papers selected for the drugs and medicines section were obtained from the Pubmed database using the following combination of words: *retina and toxicity* and *drugs*. After this first search, the search was refined using the terms *retinal toxicity* and a *specific drug*. A search with the following words did not yield any results in the last 5 years: carmustine; glitazones, rosiglitazone, and pioglitazone, as hypoglycemic agents; canthaxanthine, and methoxyflurane. The search for topical latanoprost and retina revealed only studies with protective effects. The combination of retina toxicity and the following drugs produced results: *Chloroquine, hydroxychloroquine, Phenothiazines, Pentosan polysulfate sodium, Clofazimine, Deferoxamine, Antiretroviral Therapies, Mitogen-activated protein kinase (MEK) inhibitors, Fibroblast growth factor receptor (FGFR) inhibitors, Sildenafil, Cisplatin, Aminoglycoside antibiotics, Vancomycin, Talc, Interferon, Ergot alkaloids, Gemcitabine, Epinephrine, Nicotinic acid, Niacin, Paclitaxel/docetaxel, Tamoxifen, Methanol.* Articles published in the last five years that addressed deleterious clinical and molecular aspects of the retina were considered. For textual organization, the drugs were grouped according to the clinical symptoms found in patients.
